# Society Meetings

**Published:** 1895-06

**Authors:** 


### Society Meetings.

---

THE Association of Military Surgeons of the United States held one of the most interesting meetings in its history at Buffalo, May 21, 22 and 23, 1895. The opening session was held in the Star theatre, Tuesday morning, May 21st, at which time the addresses of welcome were delivered and the president, Brigadier-General George M. Sternberg, M. D., Surgeon-General U. S. Army, pronounced the annual address.

The scientific meetings of the association were held at the University of Buffalo, beginning Tuesday afternoon and continuing during Wednesday and Thursday. On Tuesday evening an informal reception was held at the Iroquois, during which there was music and a collation served by Messrs. Wooley & Gerrans, the proprietors.

On Wednesday evening, May 22d, the doors of the Buffalo Club were thrown open to the visitors and a reception was held that did credit to the club, which is noted for its royal hospitality. Thursday, P. M., a carriage drive was given the association, which halted at Fort Porter, where the United States troops, under command of Major Daggett, were reviewed by Surgeon-General Sternberg, preceded by a brigadier-general's salute of eleven guns. In the evening a parade, review and promenade concert was given at the state arsenal by the 65th Regiment, National Guard. Friday an excursion was had to Niagara Falls. So ended an eventful meeting. The scientific work of the association is rated as above the average and a handsome, useful volume will result.

The officers for the ensuing year are: President, Louis W. Read, colonel and surgeon-general N. G. Pa., Norristown; vice-president, Albert L. Gihon, medical director U. S. N., Washington, D. C.; second vice-president, Col. Chas. H. Alden, assistant surgeon-general U. S. A.; secretary, Eustathius Chancellor, lieutenant-colonel and medical director N. G. Mo., St. Louis; treasurer, Lawrence C. Carr, major and surgeon, N. G. Ohio, Cincinnati; editor, Col. P. F. Harvey, major and surgeon U. S. A.

President-elect Read was formerly vice-president of the association. Vice-President-elect Gihon held the office of second vice-president. Secretary Chancellor and Treasurer Carr were both re-elected.

In the absence of President-elect Read, the vice-president appointed the following to serve as the executive committee: Col. Senn, ex-officio, Col. Chas. H. Alden, U. S. A., ex-officio, and Gen. J. D. Bryant, Gen. E. J. Foster, Massachusetts; Gen. Budding, Rhode Island, and Major A. H. Briggs, Buffalo.

It was decided to hold the next convention in Philadelphia.

---

THE Medical Society of the County of Erie will hold its regular semi-annual meeting on Tuesday, June 11, 1895, at the rooms of the Buffalo Academy of Medicine at 10 o'clock in the morning, under the presidency of Dr. F. W. Bartlett, of Buffalo.

